# Associated factors and predictive modeling of deep vein thrombosis in stroke patients undergoing rehabilitation

**DOI:** 10.3389/fneur.2026.1896717

**Published:** 2026-09-30

**Authors:** Yaping Wang, Weilan Jiang, Yumeng Xing, Yongjun Zheng, Jiejiao Zheng

**Affiliations:** 1 Department of Rehabilitation, Huadong Hospital, Fudan University, Shanghai, China; 2 Department of Ultrasonography, Huadong Hospital Affiliated to Fudan University, Shanghai, China

**Keywords:** associated factors, deep vein thrombosis, predictive model, rehabilitation, stroke

## Abstract

**Background:**

This study aimed to identify factors associated with deep vein thrombosis (DVT) and to develop predictive models to facilitate individualized risk assessment in this population.

**Methods:**

We retrospectively analyzed 240 patients with stroke admitted to a rehabilitation department, including 63 patients with DVT and 177 without DVT. Baseline demographic, clinical, and laboratory variables were collected. Univariate analysis was performed to screen variables potentially associated with DVT, followed by multivariable logistic regression to identify variables independently associated with DVT. In parallel, six machine-learning models were developed using all baseline variables. The full cohort was evaluated across 100 stratified 8:2 train-test splits, with repeated five-fold cross-validation performed within each training subset and corresponding held-out subsets used for testing. Model performance was assessed using the area under the receiver operating characteristic curve (AUC), and SHapley Additive exPlanations (SHAP) were used to enhance interpretability.

**Results:**

Multivariable analysis identified lower Barthel Index [odds ratio (OR) = 0.979, *p* = 0.008], diabetes (OR = 0.434, *p* = 0.032), and malignant tumor (OR = 2.883, *p* = 0.014) as variables independently associated with DVT. The final logistic regression model achieved an AUC of 0.765. Among machine learning models, the random forest model demonstrated the highest mean cross-validated AUC (0.781 ± 0.022) and the highest mean held-out AUC across 100 stratified train-test splits (0.797 ± 0.062). SHAP analysis indicated that age, D-dimer level, Barthel Index, activated partial thromboplastin time (APTT), and disease course were the five most influential contributors to Random Forest predictions.

**Conclusion:**

In conclusion, our findings support the use of integrated predictive approaches for DVT risk assessment and targeted screening in stroke rehabilitation settings.

## 1 Introduction

Stroke patients admitted to rehabilitation units face a markedly elevated risk of venous thromboembolism. The often-severe limb paralysis, prolonged bedrest, and reduced mobility common after stroke lead to venous stasis and a pro-thrombotic state (1). In addition, many stroke patients receive sedative or paralytic medications and may have systemic conditions (such as malignancy or coagulation disorders) that further increase blood coagulability (2). Altogether, these factors render stroke survivors especially prone to deep vein thrombosis (DVT) of the lower limbs.

Indeed, DVT is a frequent and often silent complication after stroke. Reported incidences vary widely depending on surveillance methods and timing. In immobilized post-stroke patients, studies have reported DVT rates from roughly 10% up to 75% (3). Although DVT is developed by up to 80% of severely impaired stroke patients in the absence of prophylaxis (2, 4), these occurrences remain largely subclinical. Systematic ultrasound screening may detect DVT in more than half of this population, yet overt symptoms or recognizable clinical signs are eventually manifested by only a small minority (1–5%) (5). Because undiagnosed DVT can have serious consequences, early evaluation and screening for DVT in stroke patients is crucial.

The paramount danger of post-stroke DVT is its potential to cause pulmonary embolism (PE). Embolic PE is a leading contributor to early mortality after stroke. Studies estimate that when a proximal limb DVT is found, roughly 30–40% of patients have already sustained a PE (6). In stroke populations, PE accounts for a substantial fraction of deaths. For example, one review notes that DVT clots leading to PE cause about 13–25% of early post-stroke deaths (7); another analysis similarly reports that PE-related deaths make up roughly 20–25% of early stroke mortality (8). Correspondingly, even a small incidence of PE has a large clinical impact: in a Canadian stroke registry (*n* ≈ 11,000), symptomatic PE occurred in approximately 0.8% of patients but was associated with a dramatic increase in short-term mortality (30-day death rate 25.8% with PE vs. 13.6% without) (9). Moreover, stroke outcomes are independently worsened by new DVT events; for instance, it was found in one analysis that significantly higher 3-month mortality was experienced by stroke patients who developed a new DVT compared to those who did not (10). In summary, DVT after stroke is a harbinger of pulmonary embolism and poor prognosis, even when initially asymptomatic.

Numerous clinical factors are known to influence DVT risk in stroke. Greater stroke severity and immobility (e.g., higher NIH Stroke Scale scores, bilateral hemiparesis, or coma) consistently elevate DVT incidence (11, 12). Additional patient factors such as female sex, older age, and pre-existing thrombophilias are also found to contribute to the overall risk (13).

Because most post-stroke DVTs are clinically silent, active screening with Doppler ultrasound is recommended in high-risk patients (14). Early identification of high-risk individuals is essential to enable timely prophylaxis and reduce the risk of fatal thromboembolism. However, no widely accepted risk stratification model exists specifically for stroke patients, particularly in the rehabilitation setting. Therefore, in this study, we retrospectively analyzed stroke patients admitted to a rehabilitation department to identify factors associated with DVT. We further constructed a multivariable logistic regression model and developed an internally evaluated machine-learning framework for DVT risk estimation, aiming to provide a robust and interpretable tool for individualized risk assessment and targeted screening in stroke rehabilitation.

## 2 Methods

### 2.1 Study population

Patients with stroke were retrospectively identified from the institutional clinical database of the Department of Rehabilitation at Huadong Hospital, Shanghai, China. This retrospective analytical study included adult inpatients with a diagnosis of stroke who received rehabilitation treatment between January 1, 2020 and November 30, 2025. Baseline demographic and clinical characteristics were extracted from electronic medical records, including variables potentially associated with DVT. DVT was diagnosed in accordance with the 2017 Chinese Guidelines for the Diagnosis and Treatment of Deep Vein Thrombosis (3rd Edition) and was defined as lower-extremity DVT detected by ultrasound within 3 days after rehabilitation admission. All ultrasound-confirmed lower-extremity DVTs were included as outcome events regardless of symptomatic status, anatomical location (proximal or distal), or laterality (unilateral or bilateral), and isolated distal DVTs were also included. Because prior serial ultrasound examinations were not routinely available, newly developed DVT could not be reliably distinguished from pre-existing asymptomatic thrombi. Therefore, the outcome reflects DVT status at early rehabilitation assessment rather than incident DVT after admission.

Patients were selected according to predefined eligibility criteria. The inclusion criteria were as follows: (1) age ≥ 18 years; (2) diagnosis of stroke according to the criteria established at the Fourth National Conference; and (3) confirmation of cerebral infarction or cerebral hemorrhage by cranial computed tomography (CT) or magnetic resonance imaging (MRI). The exclusion criteria were as follows: (1) stroke secondary to metastatic tumors or caused by primary intracranial tumors; (2) history of cranial trauma associated with the current episode; (3) history of one or more organ failure; and (4) incomplete medical records, particularly absence of ultrasound examination data.

A schematic overview of the study design, including data collection, candidate-variable screening, and machine learning model development, is presented in Figure 1.

**Figure 1:**
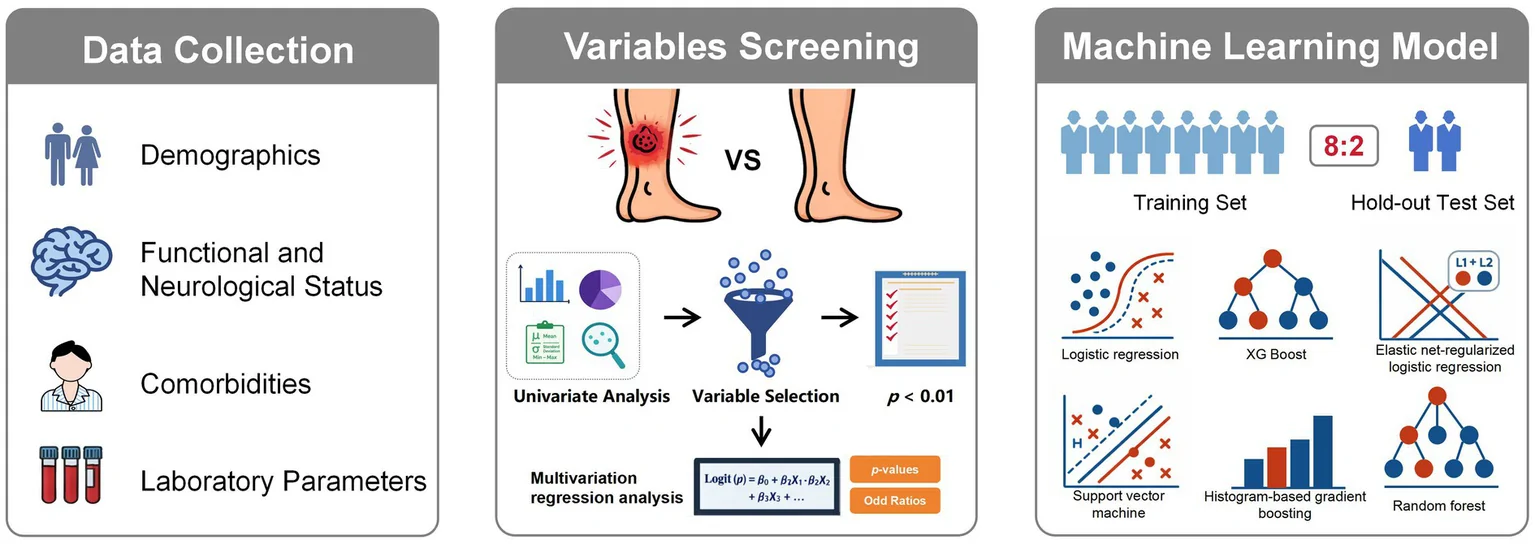
Overview of the study design and analytical workflow for identifying variables associated with DVT and developing predictive models for DVT in stroke patients undergoing rehabilitation.

### 2.2 Data collection

Demographic information collected at admission included age, sex, height, weight, body mass index (BMI), stroke diagnosis category, and disease course. Clinical variables included Brunnstrom stage of the upper limb, hand, and lower limb, Barthel Index, swallowing dysfunction, cognitive disorder, and aphasia. Information regarding comorbidities was also recorded, including hypertension, diabetes, atrial fibrillation, history of surgery or trauma, and malignant tumor. Laboratory data were obtained from blood samples collected before lower-extremity ultrasound assessment for DVT and included platelet (PLT) count, prothrombin time, international normalized ratio (INR), fibrinogen (FIB), activated partial thromboplastin time (APTT), and plasma D-dimer levels. All demographic, clinical, functional, comorbidity, and laboratory variables used in the analyses were obtained before ultrasound-based DVT assessment.

### 2.3 Identification of candidate variables for multivariable analysis

Baseline demographic, clinical, and laboratory variables were first evaluated using univariate analysis to compare patients with and without DVT and to screen for variables potentially associated with DVT. Both continuous variables (e.g., age, anthropometric measures, neurological function scores, and laboratory parameters) and categorical variables (e.g., diagnosis type, neurological complications, and comorbidities) were included in the screening process.

Variables demonstrating strong statistical significance in the univariate analysis (*p* < 0.01) were selected as candidate variables for the subsequent multivariable logistic regression analysis. This stricter threshold was applied to reduce the number of variables entered into the conventional regression model given the limited number of DVT events.

### 2.4 Logistic regression model construction

Multivariable logistic regression analysis was performed to evaluate independent associations between candidate variables and DVT. Variables that met the predefined significance criterion in the univariate analysis (*p* < 0.01) were entered into the multivariable model. This criterion identified five candidate variables, all of which were included in the multivariable analysis. With 63 DVT events, this corresponded to approximately 12.6 outcome events per included variable.

To explore the incremental contribution of candidate variables, a series of logistic regression models with progressively expanded combinations of variables were constructed. Variables were sequentially incorporated into the models according to their statistical significance in the univariate analysis and their clinical relevance. Predicted probabilities of DVT were generated for each model and subsequently used for performance evaluation as described in the statistical analysis section.

### 2.5 Machine learning model development

Machine-learning development was performed independently of the univariate variable-screening procedure used for conventional regression. All 25 prespecified baseline demographic, clinical, functional, comorbidity, and laboratory variables were included as model inputs without outcome-based univariate feature selection. To preserve the original DVT distribution and avoid distortion of the held-out evaluation cohort, no case–control matching, under sampling, or oversampling was performed. Instead, the complete cohort of 240 patients was directly divided into training and held-out test subsets using stratified random sampling at an 8:2 ratio. Each split therefore contained 192 training patients (50 with DVT and 142 without DVT) and 48 held-out patients (13 with DVT and 35 without DVT). This procedure was repeated using 100 different train-test split seeds to quantify the variability in performance attributable to data partitioning. No split was selected on the basis of held-out test performance.

Disease course, platelet count, prothrombin time, international normalized ratio, APTT, and D-dimer were log-transformed using log(1+x) , consistent with the predefined preprocessing procedure. Missing-value imputation and feature standardization were incorporated within the model-training pipeline; medians and scaling parameters were therefore estimated from the relevant training data only and subsequently applied to validation or held-out observations. Multiple machine learning algorithms were evaluated for discrimination of DVT status, including logistic regression, elastic net-regularized logistic regression, linear support vector machine (SVM), random forest, histogram-based gradient boosting, and extreme gradient boosting (XGBoost). No separate hyperparameter optimization was performed; model settings were fixed and kept unchanged across the 100 train-test partitions. Logistic regression and linear SVM used balanced class weights; elastic net logistic regression used an $l_{1}$ ratio of 0.5; linear SVM probabilities were calibrated using sigmoid calibration with five-fold cross-validation; random forest used 800 trees with balanced class weights; and XGBoost used 300 trees, a maximum depth of 3, a learning rate of 0.05, and a class-imbalance weight calculated from the training data. Histogram-based gradient boosting otherwise used its default model settings.

Within each of the 100 training subsets, model performance was evaluated using repeated stratified five-fold cross-validation with 20 repeats. Out-of-fold probabilities generated during cross-validation were used to estimate internal discrimination and to determine classification thresholds without reference to the corresponding held-out test subset. Each algorithm was then refitted using the complete training subset and evaluated once on the corresponding held-out test subset. Performance was summarized across all 100 train-test partitions.

To enhance model interpretability, SHapley Additive exPlanations (SHAP) were calculated to quantify the contribution of individual input variables to model predictions. For each of the 100 stratified splits, SHAP values were calculated only for patients in the held-out test subset. For each patient, SHAP values were subsequently averaged across the splits in which that patient had served as a held-out observation, providing a patient-level explanation based exclusively on out-of-sample predictions.

### 2.6 Statistical analysis

Continuous variables are presented as mean ± standard deviation, whereas categorical variables are summarized as counts and percentages. In the univariate analysis, continuous variables were compared between patients with and without DVT using the Mann–Whitney U test, while categorical variables were compared using the chi-square test or Fisher’s exact test, as appropriate. Logistic regression coefficients were transformed into odds ratios (ORs) with corresponding 95% confidence intervals (CIs).

For the machine learning models, discriminative performance was assessed using the area under the receiver operating characteristic curve (AUC). Sensitivity, specificity, and overall accuracy were calculated based on the confusion matrix. The optimal classification threshold was determined using Youden’s index, defined as the point on the ROC curve that maximizes the sum of sensitivity and specificity. The Youden threshold was determined from out-of-fold predictions in the training set and applied unchanged to the corresponding held-out test set. The 95% CI for the AUC was estimated using the DeLong method, whereas the 95% CIs for sensitivity, specificity and accuracy were calculated using Wilson’s score method. To account for variability arising from data partitioning, model performance was summarized across these splits using the mean and standard deviation. For overall ROC visualization, repeated out-of-fold and held-out prediction probabilities were averaged at the patient level before ROC analysis. All statistical analyses and machine learning model development were performed using Python (version 3.9).

## 3 Results

### 3.1 Baseline characteristics of the study population

A total of 240 patients with stroke were included in the study, including 63 patients with DVT and 177 patients without DVT. The baseline demographic, clinical, and laboratory characteristics of the study population are summarized in Table 1.

**Table 1:** Baseline demographic, clinical, and laboratory characteristics of patients with and without DVT.

Variable	Total (*N* = 240)	DVT (*N* = 63)	Non-DVT (*N* = 177)	*p*-value
Demographics				
Age (years)	71.85 ± 10.56	76.00 ± 7.79	70.37 ± 11.03	<0.001
Male sex, n (%)	145 (60.4)	33 (52.4)	112 (63.3)	0.136
BMI (kg/m^2^)	23.22 ± 3.25	22.10 ± 3.22	23.39 ± 3.23	0.184
Height (cm)	166.54 ± 8.87	163.64 ± 9.23	166.96 ± 8.79	0.243
Weight (kg)	64.59 ± 11.97	59.07 ± 8.65	65.39 ± 12.21	0.090
Disease course (days)	326.38 ± 791.04	203.00 ± 499.90	368.21 ± 865.07	0.317
Functional and neurological status				
Brunnstrom stage—upper limb	3.33 ± 1.63	2.91 ± 1.57	3.48 ± 1.64	0.026
Brunnstrom stage—hand	3.32 ± 1.73	2.93 ± 1.62	3.45 ± 1.75	0.059
Brunnstrom stage—lower limb	3.33 ± 1.42	2.91 ± 1.50	3.48 ± 1.37	0.011
Barthel index	43.44 ± 24.34	33.28 ± 20.66	47.27 ± 24.58	<0.001
Swallowing dysfunction	34 (14.2)	13 (20.6)	21 (11.9)	0.095
Cognitive disorder	80 (33.3)	24 (38.1)	56 (31.6)	0.348
Aphasia	98 (40.8)	25 (39.7)	73 (41.2)	0.882
Comorbidities				
Hypertension	195 (81.2)	50 (79.4)	145 (81.9)	0.708
Diabetes	87 (36.2)	13 (20.6)	74 (41.8)	0.004
History of surgery/trauma	31 (12.9)	10 (15.9)	21 (11.9)	0.512
Atrial fibrillation	28 (11.7)	8 (12.7)	20 (11.3)	0.820
Malignant tumor	39 (16.2)	18 (28.6)	21 (11.9)	0.005
Laboratory parameters				
PLT count (×10^9^/L)	222.62 ± 136.37	209.35 ± 77.26	227.37 ± 151.95	0.226
Prothrombin time (s)	12.58 ± 6.37	12.14 ± 1.22	12.74 ± 7.38	0.044
INR	1.12 ± 0.62	1.06 ± 0.10	1.14 ± 0.72	0.358
APTT (s)	30.40 ± 5.52	29.72 ± 5.41	30.64 ± 5.55	0.032
FIB (g/L)	3.41 ± 0.91	3.38 ± 0.98	3.42 ± 0.88	0.683
D-dimer (mg/L)	1.00 ± 1.29	1.37 ± 1.42	0.87 ± 1.22	<0.001

BMI, body mass index; PLT, platelet count; INR, international normalized ratio; APTT, activated partial thromboplastin time; FIB, fibrinogen; DVT, deep vein thrombosis.

Patients with DVT were significantly older than those without DVT (*p* < 0.001) and had poorer functional status, reflected by lower Brunnstrom stages of the upper limb (*p* < 0.05) and lower limb (*p* < 0.05), as well as a lower Barthel Index (*p* < 0.001). In addition, diabetes (*p* < 0.01) and malignant tumor (*p* < 0.01) were more frequently observed in patients with DVT. Among laboratory parameters, prothrombin time (*p* < 0.05), APTT (*p* < 0.05), and plasma D-dimer levels (*p* < 0.001) differed significantly between the two groups. Other variables, including sex, body mass index, hypertension, and platelet count, did not show statistically significant differences.

### 3.2 Identification of candidate variables

Univariate analysis was performed to identify variables potentially associated with DVT. Clinically relevant factors reflecting coagulation status, functional impairment, and comorbid conditions showed significant differences between patients with and without DVT. These included plasma D-dimer levels (*p* = 1.25 × 10^−4^), Barthel Index (*p* = 1.49 × 10^−4^), age (*p* = 2.38 × 10^−4^), diabetes (*p* = 0.0035), and malignant tumor (*p* = 0.0046). These variables satisfied the predefined screening criterion (*p* < 0.01) and were therefore selected as candidate variables for subsequent multivariable modeling.

Additional variables reflecting motor function and coagulation parameters, including Brunnstrom stage of the lower limb (*p* = 0.011), Brunnstrom stage of the upper limb (*p* = 0.026), APTT (*p* = 0.032), prothrombin time (*p* = 0.044), and Brunnstrom stage of the hand (*p* = 0.059), also demonstrated statistical or borderline differences between groups. These variables did not meet the predefined significance threshold (*p* < 0.01) for inclusion in the multivariable logistic regression model.

### 3.3 Logistic regression and model performance

After adjustment for other variables in the model, Barthel Index and malignant tumor remained independently associated with DVT, while diabetes also showed a statistically significant but inverse association, whereas age showed a borderline association (Table 2). Lower Barthel Index, reflecting poorer functional status, was significantly associated with an increased risk of DVT (OR = 0.979, 95% CI: 0.964–0.995, *p* = 0.008). Malignant tumor was associated with a substantially higher risk of DVT (OR = 2.883, 95% CI: 1.244–6.680, *p* = 0.014). Diabetes was also independently associated with DVT (OR = 0.434, 95% CI: 0.202–0.931, *p* = 0.032), indicating an inverse relationship that may reflect residual confounding or selection bias. Increasing age demonstrated a marginal association with DVT risk (OR = 1.039, 95% CI: 0.999–1.080, *p* = 0.053). D-dimer was not independently associated with DVT after multivariable adjustment (OR = 1.855, 95% CI: 0.879–3.916, *p* = 0.105).

**Table 2:** Multivariable logistic regression analysis of variables associated with DVT.

Variable	*B*	OR	95% CI	*p*-value
D-dimer	0.618	1.855	(0.879, 3.916)	0.105
Barthel index	−0.021	0.979	(0.964, 0.995)	0.008
Age	0.038	1.039	(0.999, 1.080)	0.053
Diabetes	−0.836	0.434	(0.202, 0.931)	0.032
Malignant tumor	1.059	2.883	(1.244, 6.680)	0.014

Regression coefficients (B), odds ratios (ORs), 95% confidence intervals (CIs), and *p*-values are shown.

ROC curves were first constructed to evaluate the discriminative ability of individual continuous variables for DVT (Figure 2A). The AUC values were 0.664 for D-dimer, 0.669 for the Barthel Index, and 0.656 for age, indicating modest discriminative performance when each variable was considered individually. To evaluate the incremental discriminative value of candidate variables, a series of logistic regression models were constructed by sequentially incorporating variables according to their statistical significance and clinical relevance (Figure 2B). The baseline model including D-dimer alone achieved an AUC of 0.664. Addition of the Barthel Index increased the AUC to 0.713, followed by further improvement with the inclusion of age (AUC = 0.722) and diabetes (AUC = 0.745). The final model incorporating D-dimer, Barthel Index, age, diabetes, and malignant tumor achieved the highest discrimination performance, with an AUC of 0.765.

**Figure 2:**
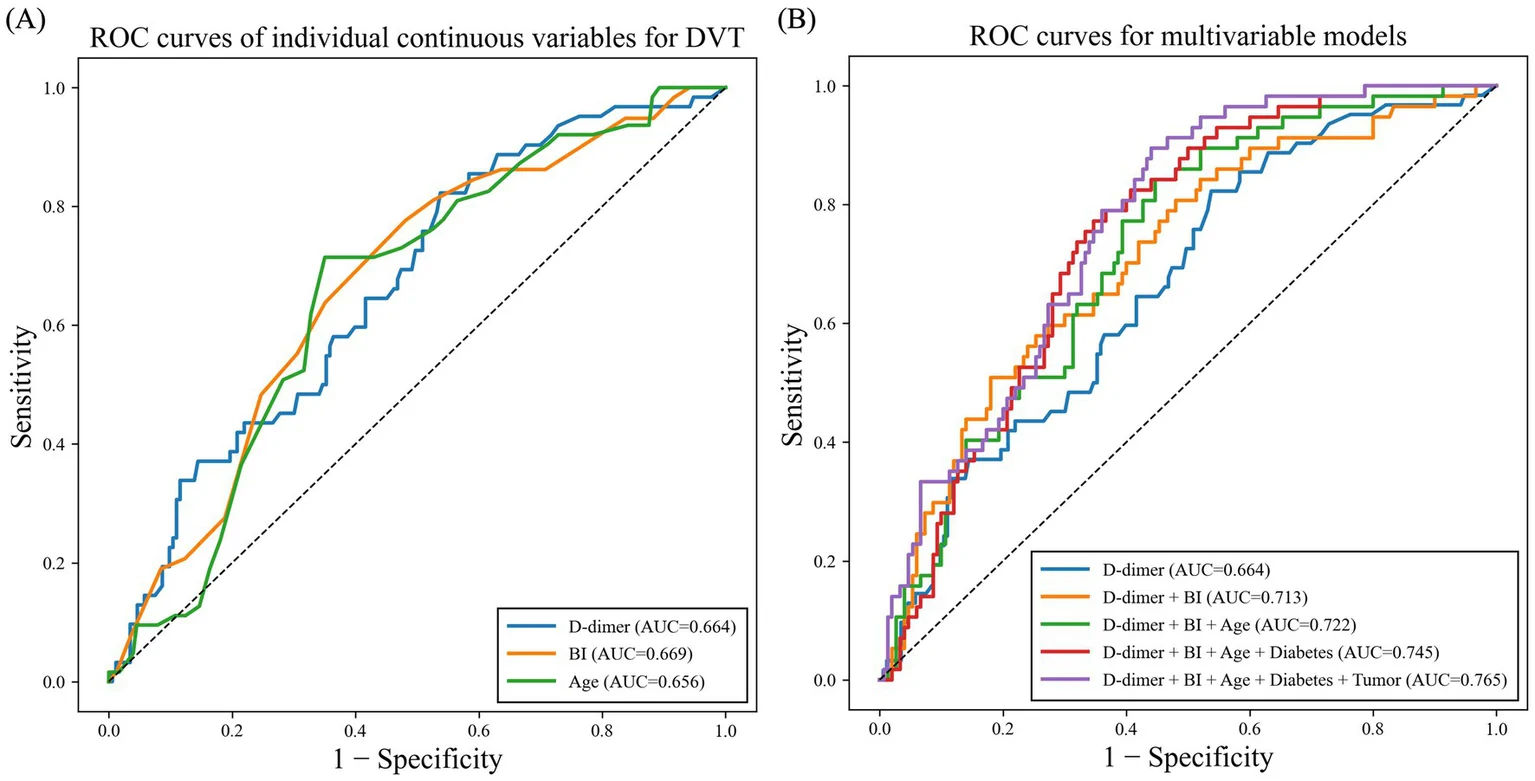
ROC curves for discrimination of DVT status using conventional logistic regression analysis. **(A)** ROC curves of individual continuous variables (D-dimer, Barthel Index, and age). **(B)** ROC curves of multivariable logistic regression models with sequential addition of candidate variables.

### 3.4 Machine learning model performance

Six machine learning algorithms were developed using all 25 prespecified baseline variables and evaluated across 100 stratified 8:2 train-test partitions. Within each training subset, model development was performed using repeated stratified five-fold cross-validation with 20 repeats. Across the 100 training partitions, the random forest model achieved the highest mean cross-validated AUC (0.781 ± 0.022), followed by XGBoost (0.755 ± 0.026) and gradient boosting (0.747 ± 0.025). Elastic net logistic regression, standard logistic regression, and linear SVM showed lower mean AUCs of 0.679 ± 0.025, 0.677 ± 0.025, and 0.672 ± 0.027, respectively.

Across the 100 corresponding held-out test subsets, the random forest model again demonstrated the highest mean AUC (0.797 ± 0.062), followed by gradient boosting (0.776 ± 0.065) and XGBoost (0.773 ± 0.069). Elastic net logistic regression and linear SVM achieved similar mean AUCs (0.698 ± 0.065 for both), while standard logistic regression achieved an AUC of 0.696 ± 0.066. Overall ROC performance is shown in Figure 3. Detailed classification metrics are presented in Table 3. Using the Youden-optimized thresholds, the random forest model achieved a mean sensitivity of 0.711 ± 0.165, specificity of 0.657 ± 0.132, and accuracy of 0.671 ± 0.077. Gradient boosting achieved the highest mean specificity (0.723 ± 0.112) and accuracy (0.703 ± 0.069), although with a lower sensitivity of 0.648 ± 0.146.

**Figure 3:**
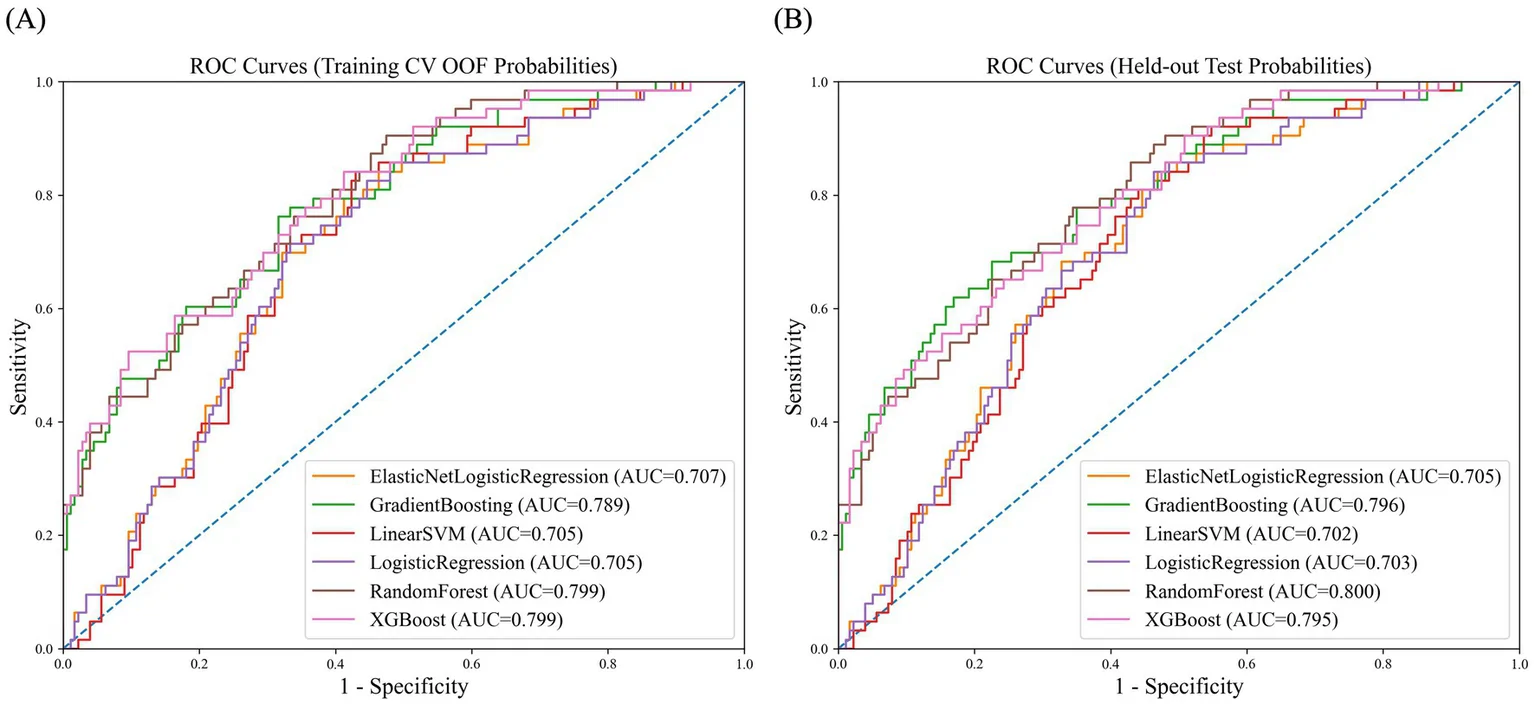
ROC curves of machine learning models for discrimination of DVT status. **(A)** ROC curves based on patient-level averaged out-of-fold probabilities generated from repeated stratified cross-validation across the training subsets. **(B)** ROC curves based on patient-level averaged held-out probabilities across the repeated internal train-test partitions. AUC values are shown in the legend and correspond to ROC analysis of the patient-level averaged probabilities.

**Table 3:** Performance of machine learning models for predicting DVT across 100 stratified held-out test evaluations.

Model	AUC	Sensitivity	Specificity	Accuracy
Logistic regression	0.696 ± 0.066	0.739 ± 0.136	0.559 ± 0.109	0.608 ± 0.070
Elastic net	0.698 ± 0.065	0.738 ± 0.142	0.562 ± 0.102	0.610 ± 0.067
Linear SVM	0.698 ± 0.065	0.735 ± 0.123	0.579 ± 0.101	0.621 ± 0.070
Random forest	0.797 ± 0.062	0.711 ± 0.165	0.657 ± 0.132	0.671 ± 0.077
Gradient boosting	0.776 ± 0.065	0.648 ± 0.146	0.723 ± 0.112	0.703 ± 0.069
XGBoost	0.773 ± 0.069	0.688 ± 0.138	0.667 ± 0.116	0.673 ± 0.073

Values are presented as mean ± standard deviation.

Overall, ensemble-based models consistently outperformed linear models in terms of discriminative ability, with the random forest model achieving the highest mean AUC across the repeated held-out test evaluations.

### 3.5 Model interpretability analysis

To enhance model interpretability, SHAP analysis was applied to the Random Forest algorithm, which achieved the highest mean cross-validated AUC across the 100 data partitions. As shown in Figure 4A, age showed the greatest mean absolute SHAP value, followed by plasma D-dimer, Barthel Index, APTT, and disease course. Diabetes and prothrombin time also made substantial contributions, followed by body mass index, weight, platelet count, height, and fibrinogen. Brunnstrom motor stages contributed more modestly, whereas malignant tumor and several other categorical variables showed lower global SHAP importance.

**Figure 4:**
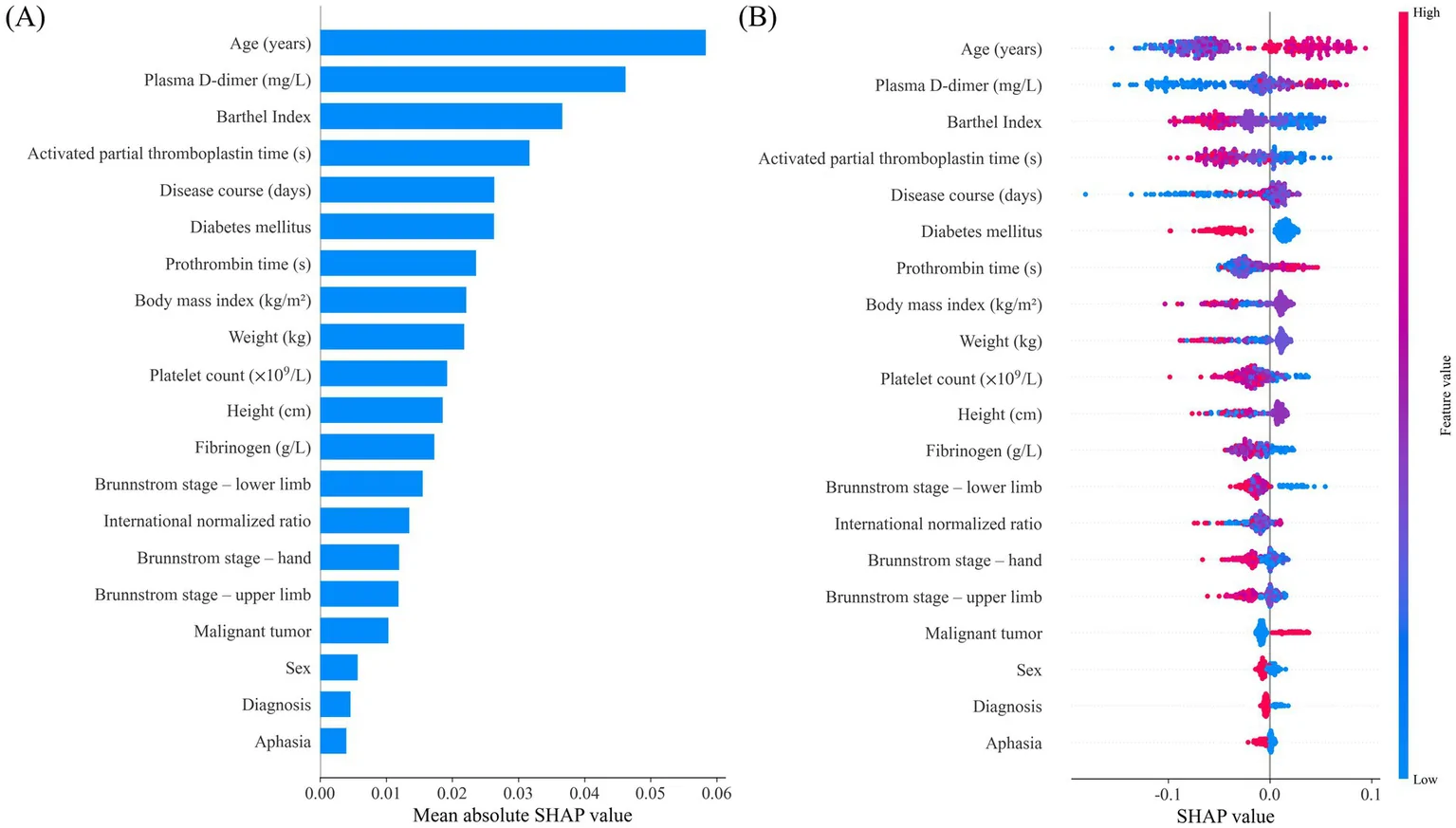
SHAP-based interpretation of the Random Forest model for discrimination of DVT status. **(A)** The top 20 input variables ranked by mean absolute SHAP values. **(B)** SHAP summary (beeswarm) plot showing the distribution and direction of feature contributions to model predictions. Each point represents one patient, and color indicates feature value (blue: low; red: high). Patient-level SHAP values were derived from held-out predictions and averaged across the splits in which each patient served as a held-out observation.

The SHAP summary plot (Figure 4B) illustrates the distribution and direction of feature contributions across patients. Higher age and elevated plasma D-dimer levels were consistently associated with increased predicted DVT risk, whereas higher Barthel Index was associated with lower predicted risk. Better Brunnstrom motor stages also generally contributed toward lower predicted risk, although their overall importance was smaller. Higher APTT values tended to contribute toward lower predicted risk, whereas lower values more often contributed positively. Disease course showed a more heterogeneous pattern, consistent with a potentially non-linear association within the Random Forest model. The contribution of diabetes appeared predominantly toward lower predicted risk, consistent with the multivariable regression results, whereas malignant tumor tended to increase predicted risk. Higher prothrombin time also tended to contribute toward higher model-predicted DVT risk.

These results indicate that the Random Forest model integrates demographic characteristics, coagulation biomarkers, comorbidities, and functional status indicators to estimate DVT risk, capturing predictive patterns that are not restricted to simple linear associations. SHAP values should, however, be interpreted as contributions to model predictions rather than as estimates of independent causal effects.

## 4 Discussion

Our data provide evidence that lower functional status (as reflected by a lower Barthel Index) and concurrent malignancy are independently associated with lower-limb DVT in stroke patients entering rehabilitation, whereas age demonstrated only a borderline association after adjustment. These findings reinforce that stroke survivors, particularly those with impaired mobility, are prone to thrombotic complications (15). From a pathophysiological perspective, Virchow’s triad of stasis, endothelial injury, and hypercoagulability explains thrombogenesis (16). Factors such as advanced age, impaired functional status, diabetes, and malignancy may promote DVT by these mechanisms. In agreement with prior reports, our findings showed associations of impaired functional status and malignancy with DVT (2). Specifically, aging is associated with vascular endothelial dysfunction and increased coagulation factors (17), while reduced mobility further promotes venous stasis (16). Diabetes may additionally induce a prothrombotic state via endothelial damage and altered coagulation (18), whereas malignancy induces hypercoagulability through tumor-secreted procoagulant factors (19). In our logistic model, each one-point increase in Barthel Index was associated with a reduced risk of DVT, indicating an inverse association between DVT and better functional independence. Malignant tumor was associated with an approximately 2.9-fold increase in DVT odds (OR = 2.883). Diabetes was also independently associated with DVT (OR = 0.434, 95% CI: 0.202–0.931, *p* = 0.032), although the observed inverse relationship may reflect residual confounding or selection bias rather than a true protective effect. Age showed only a marginal association with DVT risk (*p* = 0.053), suggesting that the association was attenuated after adjustment for functional status and other included variables. Notably, the repeated held-out Random Forest SHAP analysis showed that age, plasma D-dimer, Barthel Index, APTT, and disease course contributed most strongly to model predictions, with additional contributions from diabetes and prothrombin time. Unlike the conventional regression analysis, the machine-learning models included all prespecified baseline variables irrespective of their univariate statistical significance. The differing rankings from the two analytical approaches should therefore be interpreted in light of their distinct purposes: multivariable logistic regression estimates adjusted associations under a specified parametric form, whereas SHAP values quantify how strongly individual variables contribute to predictions generated by the fitted Random Forest. A variable may therefore have high predictive importance despite lacking a statistically significant independent regression coefficient. Conversely, SHAP importance does not establish an independent causal association.

The factors associated with DVT in our analysis align in part with prior studies of post-stroke DVT. For example, a recent investigation of acute stroke patients (onset under 14 days) found that older age, lower Berg Balance Scale (BBS) scores, and higher D-dimer independently predicted DVT (20). In comparison, our cohort encompassed not only acute-phase patients but also those in recovery and chronic (sequelae) stages. To our knowledge, this is the first study to analyze factors associated with DVT across the full spectrum of stroke rehabilitation (acute through chronic) and to employ both classical regression and machine-learning approaches, thereby extending previous work that was largely limited to acute-phase populations. Consistent with Li et al., age showed an association with DVT in the univariate analysis, although this association was only borderline after multivariable adjustment in our cohort. In contrast to the Berg Balance Scale used in their study, the Barthel Index, a global measure of daily function, remained significantly associated with DVT in our multivariable analysis. We did not find D-dimer to be independently associated with DVT in multivariate analysis (OR = 1.855, *p* = 0.105), likely reflecting confounding by other clinical factors and variability in measurement timing, despite its established role as a thrombotic biomarker; however, its relatively high importance in the SHAP analysis indicates that D-dimer nevertheless provided substantial information to Random Forest predictions. This predictive contribution may reflect nonlinear or conditional patterns not represented by a single adjusted linear coefficient. The inclusion of patients with a broad range of post-stroke durations extends the population examined beyond predominantly acute-phase cohorts; however, stage-specific DVT risk was not evaluated in the present study. The use of ML models demonstrated predictive contributions from these and additional variables, warranting further investigation.

Deep venous thrombosis is a serious contraindication for intensive rehabilitation, as an undiagnosed clot can embolize and dramatically worsen outcomes (21). In our rehabilitation population, the DVT incidence was approximately 26% (63 of 240 patients). This falls within the wide range reported in the literature, from approximately 10 to 75% in immobilized stroke patients, reflecting differences in screening methods and patient populations (22). Given the high stakes, simple and reliable screening is essential prior to rehabilitation. Several risk-assessment tools exist. The Wells score is convenient (few items) and performs well for proximal leg DVT (with an AUC around 0.75) (23), but it was developed for outpatients and largely focuses on clinical signs. As a result, it often misses isolated distal DVT, which are common and typically asymptomatic in stroke patients. The Caprini RAM (as a cumulative point system) has shown high predictive accuracy for post-stroke DVT when combined with laboratory markers (22), but it includes many variables (up to 39 in the full version), making it cumbersome for routine use. The Padua Prediction Score was designed for general medical inpatients and assigns points for 11 risk factors (active cancer, prior venous thromboembolism, reduced mobility, etc.) (24). While Padua stratifies risk of venous thromboembolism in hospitalized patients, its validity specifically in stroke rehab patients has not been established. These limitations highlight the lack of a simple and reliable DVT prediction model tailored to stroke rehabilitation populations. In this context, our study may help clinicians recognize high-risk patients earlier by quantifying incidence and identifying key variables. Improved awareness and targeted use of imaging (e.g., ultrasound) or prophylaxis in patients with advanced age, poor function, or malignancy could reduce undiagnosed DVT and thereby improve stroke outcomes.

Our study has several limitations. It was a single-center retrospective analysis with a moderate sample size, which may limit generalizability. Although the machine-learning analysis was repeated across 100 stratified train-test partitions, it represents internal repeated holdout validation rather than external validation. The relatively small held-out subsets and repeated use of patients across partitions may contribute to variability in performance estimates. For the conventional regression analysis, five variables were evaluated among 63 DVT events, corresponding to approximately 12.6 outcome events per variable. In contrast, the available sample size was relatively limited for machine-learning development using 25 input variables. Although repeated cross-validation and repeated holdout evaluation were used to assess performance stability, these procedures do not increase the effective sample size, and some degree of overfitting or model instability cannot be excluded. Accordingly, the machine-learning findings should be regarded as exploratory and require confirmation in larger independent multicenter cohorts before clinical implementation. Beyond these modeling considerations, limitations also existed in outcome ascertainment and clinical data availability. DVT assessment was based on lower-limb ultrasound performed within the first 3 days after rehabilitation admission. Accordingly, the present outcome primarily reflects DVT status at early rehabilitation assessment rather than incident DVT developing during the subsequent rehabilitation period. Because prior serial ultrasound examinations were generally unavailable, newly developed DVT could not be reliably distinguished from pre-existing asymptomatic thrombi, and late-onset DVTs occurring after the initial assessment may have been missed. DVT occurrence was also not analyzed separately according to acute, recovery, or chronic post-stroke stage; therefore, no conclusions regarding differences in DVT risk across disease stages can be drawn from the present study. In addition, some patients, particularly those with relatively mild deficits or longer post-stroke duration, declined lower-limb ultrasound screening. Their exclusion may have introduced selection bias if the probability of undergoing ultrasound was related to underlying DVT risk. Furthermore, we did not include anticoagulant or antiplatelet therapy in the analyses because medication exposure could not be reliably characterized retrospectively owing to substantial variation in treatment initiation, duration, and timing relative to DVT assessment. Future multicenter prospective studies with serial screening will be important to validate and extend these findings. Comprehensive data collection, including medication use and imaging timing, will help refine risk models and improve DVT prevention in stroke rehabilitation.

In conclusion, this study identified impaired functional status and malignancy as key factors associated with DVT, while age showed only a borderline association in multivariable regression but remained an important contributor to machine-learning predictions, in stroke patients undergoing rehabilitation across different disease stages. By integrating conventional statistical modeling with a leakage-controlled machine-learning framework evaluated across repeated stratified train-test partitions, our findings provide complementary insights into both adjusted associations with DVT and multivariable predictive patterns. The proposed modeling framework may facilitate more accurate risk stratification and support targeted screening and preventive strategies in clinical practice.

## Data Availability

The original contributions presented in the study are included in the article/supplementary material, further inquiries can be directed to the corresponding authors.
